# Cuban Sugar Cane Wax Acid and Policosanol Showed Similar Atheroprotective Effects with Inhibition of LDL Oxidation and Cholesteryl Ester Transfer via Enhancement of High-Density Lipoproteins Functionality

**DOI:** 10.1155/2019/8496409

**Published:** 2019-02-24

**Authors:** Kyung-Hyun Cho, Myung-Ae Bae, Jae-Ryong Kim

**Affiliations:** ^1^Department of Medical Biotechnology, Yeungnam University, Gyeongsan 712-749, Republic of Korea; ^2^Research Institute of Protein Sensor, Yeungnam University, Gyeongsan 712-749, Republic of Korea; ^3^LipoLab, Yeungnam University, Gyeongsan 712-749, Republic of Korea; ^4^Drug Discovery Platform Technology Team, Korea Research Institute of Chemical Technology, Taejon 305-343, Republic of Korea; ^5^Department of Biochemistry and Molecular Biology, Smart-Aging Convergence Research Center, College of Medicine, Yeungnam University, Daegu 705-717, Republic of Korea

## Abstract

**Background:**

Cuban sugarcane wax acids (SCWA) and policosanol (PCO) are mixtures of higher aliphatic acids and alcohols, respectively, purified from sugarcane wax with different chief components. Although it has been known that they have antioxidant and anti-inflammatory activities, physiological properties on molecular mechanism of SCWA have been less studied than PCO.

**Methods:**

In this study, we compared antiatherogenic activities of SCWA and PCO via encapsulation with reconstituted high-density lipoproteins (rHDL).

**Results:**

After reconstitution, SCWA-rHDL showed smaller particle size than PCO-rHDL with increase of content. PCO-rHDL or SCWA-rHDL showed distinct inhibition of glycation with similar extent in the presence of fructose. PCO-rHDL or SCWA-rHDL showed strong antioxidant activity against cupric ion-mediated oxidation of low-density lipoproteins (LDL), and inhibition of oxLDL uptake into macrophages. Although PCO-rHDL showed 1.2-fold stronger inhibition against cholesteryl ester transfer protein (CETP) activity than SCWA-rHDL, SCWA-rHDL enhanced 15% more brain cell (BV-2) growth and 23% more regeneration of tail fin in zebrafish.

**Conclusion:**

PCO and SCWA both enhance the beneficial functions of HDL to maximize its antioxidant, antiglycation, and antiatherosclerotic activities and the inhibition of CETP. These enhancements of HDL functionality by PCO and SCWA could exert antiaging and rejuvenation activity.

## 1. Introduction

Dyslipidemia is global health risk causing cardiovascular disease (CVD), the first leading cause of death in the world. A higher level of serum HDL-cholesterol is inversely correlated with the incidence of the CVD and hypertension [1, 2]. Inhibition of cholesteryl ester transfer protein (CETP) is an effective approach to raising HDL-C level and reducing major coronary events with 15% relative risk reduction [3, 4]. Besides HDL-C level in quantity, both HDL quality and HDL functionality were established as important for suppressing the incidence of metabolic syndrome [5, 6]. Antioxidant and anti-inflammatory activities of HDL are major functionalities to prevent atherogenesis, which is initiated by LDL oxidation and subsequent phagocytosis into macrophages [7]. The prevalence of dysfunctional HDL in serum is associated with greater incidence of CVD; therefore, enhancement of HDL functionality has been suggested as a potent therapeutic approach to reduce cardiovascular risk [1, 8].

In our previous studies, policosanol (PCO) has been found to have potent cardioprotective properties based on molecular basis, such as CETP inhibitory activity, antiglycation, and anti-inflammatory activities [9]. In animal studies, PCO supplementation improved dyslipidemia in zebrafish [10] and hypertension in SHR [11] with amelioration of hepatic inflammation. In human study, policosanol (PCO) supplementation raised serum HDL-C and enhanced HDL functionality to inhibit oxidation and glycation of LDL and HDL as well as lowering blood pressure in a dose-dependent manner [12–14].

PCO is a mixture of aliphatic alcohols ranging from 24 to 34 carbon atoms refined from sugar cane wax (*Saccharum officinarum* L.), namely, octacosanol, triacontanol, and dotriacontanol, hexacosanol, and tetratriacontanol as major components [15, 16].

Sugar cane wax acid (SCWA) is a mixture of 13 aliphatic primary alcohols C24, C25, C26, C27, C28, C29, C30, C31, C32, C33, C34, C35, and C36 (total purity> 75%), which is purified from saponification of sugar cane wax after extraction with n-hexane, ethanol, and acetone. SCWA, also called D-003, is the subject of fewer reports because it was developed later than PCO [17]. It has been known that D-003 inhibits cyclooxygenase activity, lipid peroxidation, and platelet aggregation [18–21].

Although SCWA or D-003 has no toxicity in long-term consumption, there is insufficient information about its effects on lipoprotein metabolism, especially in HDL functionality. In the current study, we compared the* in vitro* effects of SCWA and PCO in terms of lipoprotein functionality on the basis of molecular level.

## 2. Materials and Methods

### 2.1. Materials

Policosanol and sugar cane wax acids were obtained from Rainbow & Nature Pty, Ltd. (Thornleigh, Australia). Policosanol (PCO) contains alcohols of 8 long-chain wax alcohols, including 1-tetracosanol, 1-heptacosanol, 1-nonacosanol, 1-dotriacontanol, 1-hexacosanol, 1-octacosanol, 1-triacontanol, and 1-tetratriacontanol. SCWA contains 13 wax acids: C_24_ (tetracosanoic acid), C_25_ (pentacosanoic acid), C_26_ (hexacosanoic acid), C_27_ (heptacosanoic acid), C_28_ (octacosanoic acid), C_29_ (nonacosanoic acid), C_30_ (triacontanoic acid), C_31_ (hentriacontanoic acid), C_32_ (dotriacontanoic acid), C_33_ (tritriacontanoic acid), C_34_ (tetratriacontanoic acid), C_35_ (pentatriacontanoic acid), and C_36_ (hexatriacontanoic acid) wherein octacosanoic (C_28_) acid, an active metabolite of octacosanol, is the most bountiful compound.

### 2.2. Synthesis of Reconstituted HDL

To surmount the insolubility of PCO and SCWA in water, we synthesized rHDL containing PCO (PCO-rHDL) or SCWA (SCWA-rHDL). Reconstituted HDL (rHDL) containing either PCO or SCWA was prepared by the sodium cholate dialysis method, as in our previous report [22], using initial molar ratios of 95:5:1:1 and 95:5:1:5 for POPC: cholesterol: apoA-I: PCO or SCWA as described previously [9].

### 2.3. Fluorospectroscopy

Movement of tryptophan residues in the PCO-rHDL and SCWA-rHDL was determined from uncorrected spectra obtained on an LS55 spectrofluorometer (Perkin-Elmer, Norwalk, CT) and WinLab software package 4.00 (Perkin-Elmer) using a 1-cm path length Suprasil quartz cuvette (Fisher Scientific, Pittsburg, PA). The wavelengths of maximum fluorescence (WMF) in each rHDL were excited at 295 nm to avoid tyrosine fluorescence, and the emission spectra were scanned from 305 to 400 nm at room temperature.

### 2.4. Purification of Human Lipoprotein

Human LDL (1.019<d<1.063) were isolated via sequential ultracentrifugation from the sera of young human males (mean age, 22±2 years old) who voluntarily donated blood after fasting overnight. The density was appropriately adjusted by addition of NaCl and NaBr as standard protocols [23]. Samples were centrifuged for 24 h at 10°C at 100,000g using a Himac CP-100 NX (Hitachi, Tokyo, Japan).

### 2.5. Inhibition of LDL Oxidation

In order to compare the extent of oxidation, purified human LDL was incubated at 37°C with each rHDLs under presence of 10 *μ*M CuSO_4_. During incubation, the quantity of conjugated dienes in the form of oxidized product was monitored by measuring the absorbance at 234 nm (Abs_234_) [24] using a Beckman DU 800 spectrophotometer (Fullerton, CA, USA) equipped with a MultiTemp III thermocirculator (Amersham, Uppsala, Sweden).

To verify the spectroscopic data, oxidized samples were subjected to 0.5% agarose gel electrophoresis in order to compare electromobilities and visualized by 0.125% Coomassie Brilliant Blue staining.

### 2.6. Inhibition of HDL Glycation

To compare the inhibitory ability of PCO and SCWA against fructose-mediated glycation, SCWA-rHDL and PCO-rHDL were incubated with D-fructose (final 250 mM in the mixture). To mimic physiological condition, the reaction mixture was incubated in 5% CO_2_ at 37°C. The advanced glycated end products were determined from fluorometric intensities measured at 370 nm (excitation) and 440 nm (emission) using the LS55 spectrofluorometer (Perkin-Elmer), as described previously [25]. After incubation, in order to compare electromobilities and multimerization patterns, the samples were subjected to 15% SDS-PAGE and the Coomassie Brilliant Blue staining [26].

### 2.7. Cholesteryl Ester Transfer Inhibition Assay

An apoA-I-rHDL containing cholesteryl ester was synthesized as previously described [27] with [^3^H]-cholesteryl oleate (TRK886, 3.5 *μ*Ci/mg of apoA-I; GE Healthcare). The CE-transfer reaction was conducted in 300-*μ*L reaction mixtures each containing human HDL_3_ (20 *μ*l, 2 mg/ml) as a cholesteryl ester transfer protein (CETP) source, the apoA-I-rHDL (20 *μ*l, 0.25 mg/ml) as a CE-donor, and the human LDL (20 *μ*l, 0.25 mg/ml) as a CE-acceptor. PCO-rHDL or SCWA-rHDL was provided to the reaction mixture as an inhibitor. The extent of CETP inhibition was calculated as follows:

(1) %  inhibition=100×1−sample cpm−blank cpmcontrol cpm−blank cpm,

where the sample is rHDL containing PCO or SCWA treated as an inhibitor source, and the control is without inhibitor.

### 2.8. Uptake of oxLDL into Macrophages

Human monocyte THP-1 cells were maintained in RPMI-1640 medium (Hyclone, Logan, UT, USA) supplemented with 10% fetal bovine serum (FBS). To induce differentiation into macrophages, monocyte cells that had undergone no more than 20 passages were incubated in medium containing phorbol 12-myristate 13-acetate (PMA; final concentration of 150 nM) in 24-well plates for 48 h at 37°C in a humidified incubator (5% CO_2_ and 95% air) as our previous report [9].

The differentiated and adherent macrophages were then rinsed with warm PBS and incubated with 400 *μ*l of fresh RPMI-1640 medium containing 1% FBS, 50 *μ*l of oxLDL (50 *μ*g of protein in PBS), and either SCWA-rHDL or PCO-rHDL for 48 h at 37°C in a humidified incubator. After incubation, the cells were stained with oil red O solution (0.67%) to visualize the amounts of lipid species after uptaken. In order to determine amount of oxidized species, the cell media (0.25 ml) were then analyzed by TBARS assay using malondialdehyde (MDA) standard.

### 2.9. Cell Growth and Apoptosis

Microglial cells in human brain (BV-2) were cultured in a Dulbecco's modified Eagle medium (DMEM) and maintained at 70% confluency as our previous report [10]. Cells were treated with either PCO-rHDL or SCWA-rHDL and incubated for 48 h at 37°C in a humidified incubator (5% CO_2_ and 95% air). After incubation, the cell number was counted using Cellometer K2 image cytometer (Nexcelcom Biosciences, Lawrence, MA)

### 2.10. Zebrafish

Wildtype zebrafish (AB strain) and its embryos were maintained according to standard protocols as in our previous report [9, 10]. The maintenance of zebrafish was approved by the Committee of Animal Care and Use of Yeungnam University (Gyeongsan, Korea). The fish were kept breeding in a system cage at 28°C under a 12:12 h light cycle with consumption of Tetrabit (Tetrabit Gmbh D49304, Melle, Germany) as normal diet.

### 2.11. Fin Regeneration

In order to compare ability for tissue regeneration by PCO or SCWA, fins were tested using an STZ-induced adult zebrafish as in our previous reports [9, 10, 28]. Experimental zebrafish (11 weeks old) were anesthetized by submersion in 2-phenoxyethanol (Sigma P1126; St. Louis, MO) in system water (1:1000 dilution). After amputation, 10 *μ*L of rHDL alone (7 *μ*g of apoA-I), PCO-rHDL, or SCWA-rHDL was injected into the tail muscle of each zebrafish near the urostyle (n=9 for each group). After the injection, the fish were maintained in a 28°C system incubator. Images of regenerating fins of live zebrafish were taken at 24-h intervals for up to 168 h under a stereomicroscope (Motic SMZ 168; Hong Kong) using a Motic cam 2300 CCD camera with Image-Pro Plus software version 4.5.1.22 (Media Cybernetics, Bethesda, MD, USA).

### 2.12. Statistical Analysis

All data were expressed as the mean±SD of at least three independent experiments with duplicate samples. Data were evaluated via one-way analysis of variance (ANOVA) using SPSS (version 14.0; SPSS, Inc., Chicago, IL, USA), and the differences between the means were assessed using Duncan's multiple-range test. Statistical significance was defined as* p*<0.05.

## 3. Results

### 3.1. Characterization of PCO-rHDL and SCWA-rHDL

**Figure 1:**
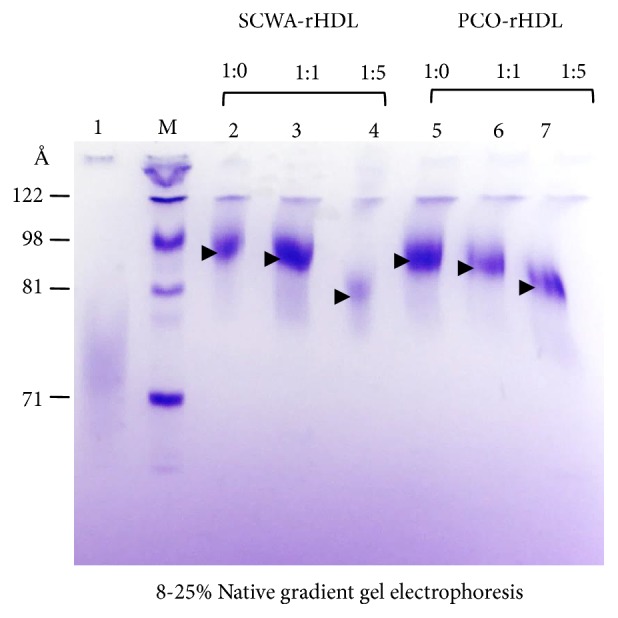
Electrophoretic patterns of rHDL by native electrophoresis (8-25% native polyacrylamide gradient gel) after synthesis of reconstituted HDL with apoA-I and either PCO or SCWA. Lane 1, lipid-free apoA-I; lane M, molecular weight marker; lane 2, SCWA-rHDL (1:0); lane 3, SCWA-rHDL (1:1); lane 4, SCWA-rHDL (1:5); lane 5, PCO-rHDL (1:0); lane 6, PCO-rHDL (1:1); lane 7, PCO-rHDL (1:5).

SCWA-rHDL and PCO-rHDL were synthesized (Figure 1) with a size of around 85-98 Å as indicated by the black arrowhead, whereas lipid-free apoA-I was about 57-68 Å in size (Table 1). Native gel electrophoresis revealed that the particle size of rHDL was reduced with increasing PCO or SCWA content (Figure 1). As its molar ratio in rHDL increased, SCWA-rHDL had a smaller particle size (85-95 Å) than PCO-rHDL (87-96 Å).

Fluorospectroscopy measurement revealed that lipid-free and lipid-bound apoA-I showed WMF of 341 and 337 nm, respectively, suggesting that Trp in apoA-I moved to a more hydrophobic phase via interaction with phospholipids as shown in Table 1. As the PCO or SCWA content of rHDL increased, WMF of apoA-I in HDL slightly shifted to red fluorescence (340 and 341 nm at molar ratios of 1:1 and 1:5, respectively). This result indicates that Trp of apoA-I in PCO-rHDL moved to a more hydrophilic phase due to interactions with PCO and exposure to the hydrophobic phase of apoA-I.

### 3.2. Antioxidant Activity of PCO and SCWA

**Figure 2:**
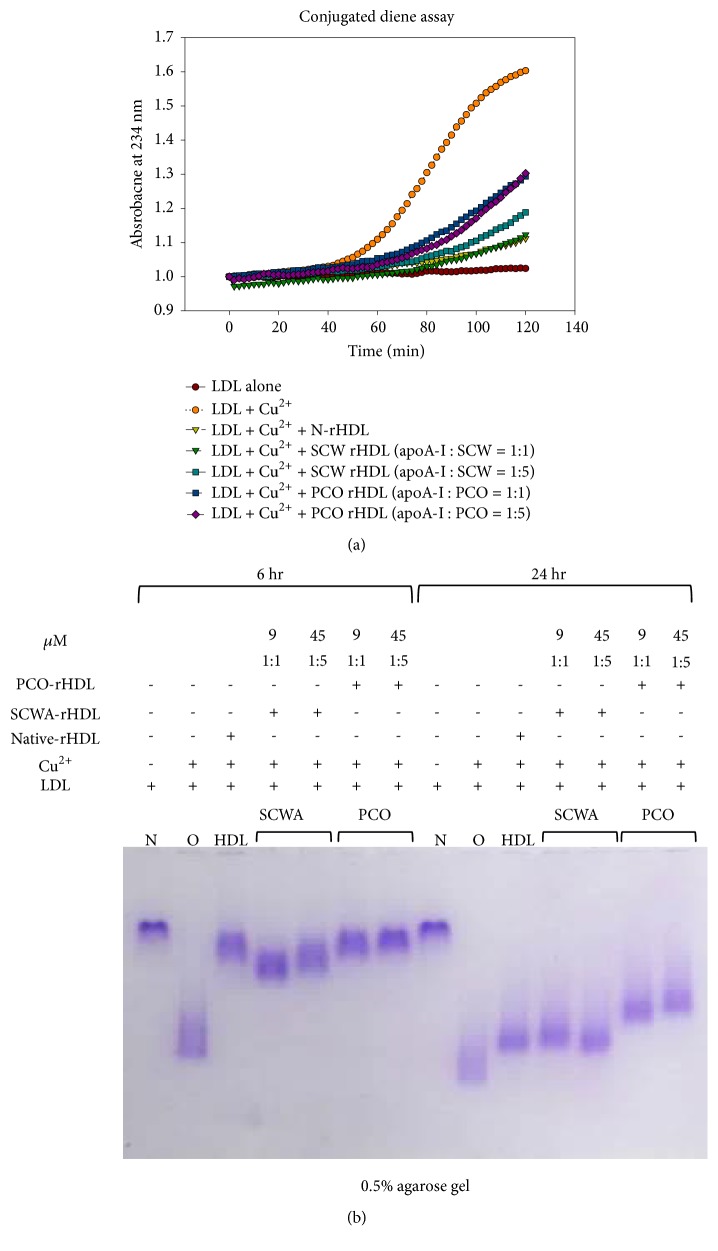
(a) Monitoring of conjugated diene production in LDL (absorbance at 234 nm) in the presence of rHDL containing either policosanol (PCO-rHDL) or SCWA (SCWA-rHDL). (b) Comparison of electromobility of LDL under presence of cupric ion and each rHDL for 6 hr and 24 hr incubation.

Conjugated diene detection assay revealed that SCWA-rHDL and PCO-rHDL showed similar antioxidant activity against cupric ion-mediated LDL oxidation after 2 h incubation (Figure 2(a)).

After 6 h incubation with cupric ion, oxidized LDL showed the fastest electromobility on 0.5% agarose gel (lane O, Figure 2(b)) compared with native LDL (lane N, Figure 2(b)). However, SCWA-rHDL or PCO-rHDL-treated LDL showed slower electromobility, indicating much less oxidation. Interestingly, after 24 h incubation in the presence of Cu^2+^, PCO-rHDL retained more antioxidant activity than SCWA-rHDL-treated LDL and showed a more distinct band with slower mobility.

### 3.3. Antiglycation Activity of PCO and SCWA

**Figure 3:**
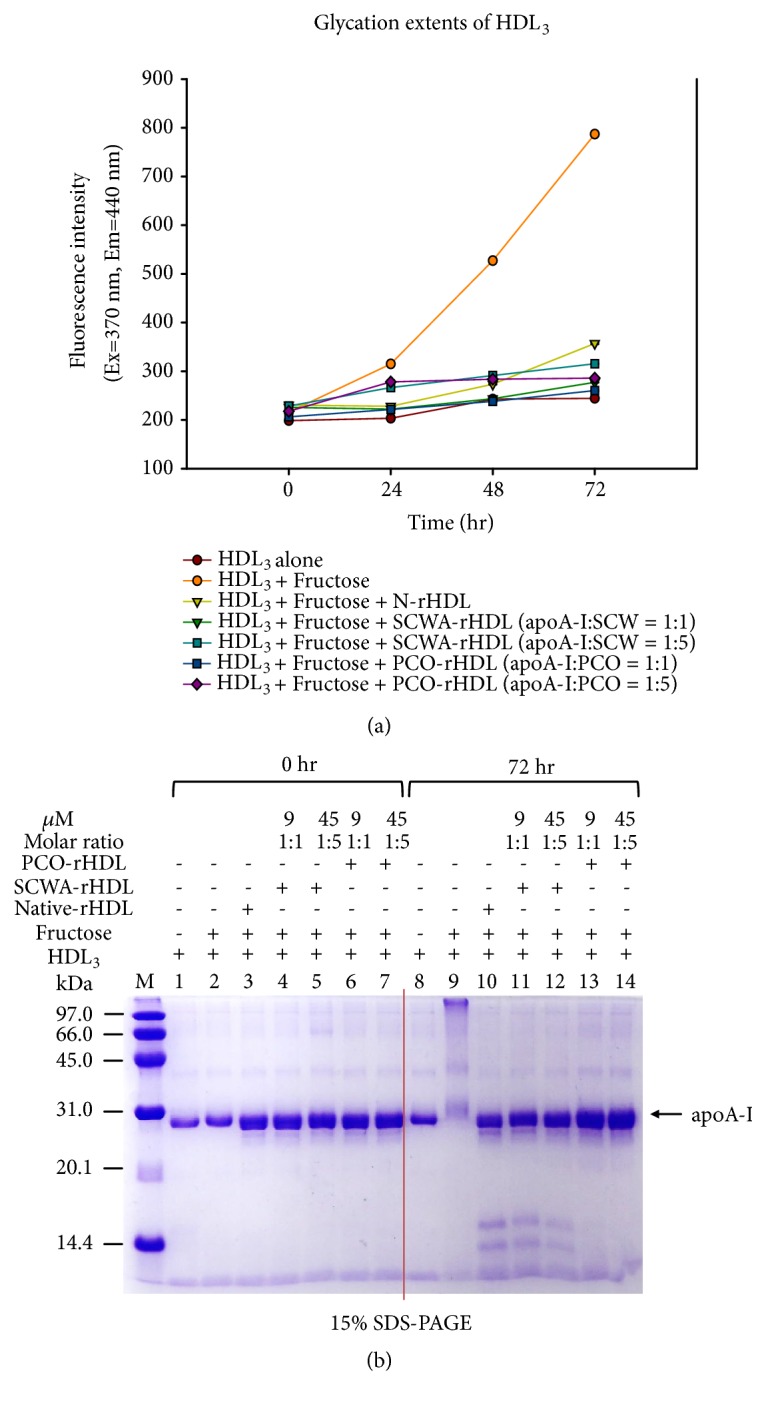
(a) Production of advanced glycation end products in HDL_3_ by fructose treatment under presence of PCO-rHDL or SCWA-rHDL. (b) Electrophoretic patterns of HDL_3_ after the fructose-mediated glycation (15% SDS-PAGE).

Glycation of HDL by fructose treatment causes dysfunctional HDL production. Fructose-treated HDL_3_ showed a 3.4-fold increase in glycation compared to HDL alone based on yellow fluorescence (Figure 3(a)).

After 72 h incubation, fructose-treated HDL_3_ showed a smeared apoA-I band pattern with severe multimerization (lane 9, Figure 3(b)), whereas native apoA-I showed a single band (lane 8, Figure 3(b)). In the presence of fructose, PCO and SCWA treatment prevented the glycation in a dose-dependent manner. Treatment with PCO-rHDL resulted in ~67% inhibition of glycation (Figure 3(a)) with a more distinct apoA-I band pattern (lanes 13, 14, Figure 3(b)). SCWA-rHDL also showed up to 60-65 % inhibition (Figure 3(a)) with a distinct apoA-I band (lanes 11, 12, Figure 3(b)). Interestingly, native-rHDL and SCWA-rHDL showed similar patterns in terms of two small fragment bands produced after 72 h incubation (lane 10-12, Figure 3(b)), while PCO-rHDL treatment did not (lane 13,14). These results suggest that the antiglycation effect of PCO and SCWA was similar due to their association with rHDL via putative protection from Lys and Arg modification induced by fructosylation, which is involved in the Maillard reaction of glycation and protein multimerization.

### 3.4. PCO and SCWA Inhibits CETP Activity

**Figure 4:**
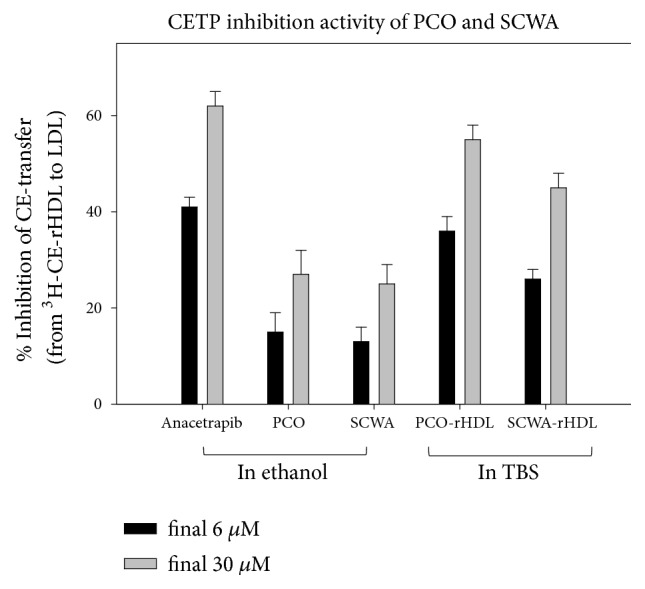
CETP inhibition activity of policosanol (PCO) or sugar cane wax acid (SCWA) in ethanol and rHDL. Data are expressed as mean ± SD of three independent experiments performed in duplicate. CE transfer from [^3^H]-HDL (50 *μ*g of apoA-I, 30,000 CPM) to human LDL (50 *μ*g of protein) by human HDL_3_ (25 *μ*g of protein) was inhibited by rHDL containing policosanol. TBS, tris-buffered saline.

Anacetrapib (MK-0859, Merck), a well-known CETP inhibitor, resulted in 41% and 62% inhibition* in vitro *at 6 and 30 *μ*M final concentrations in EtOH, respectively. In ethanol, SCWA and PCO (6 and 30 *μ*M) also resulted in approximately 15% and 26% inhibition, respectively (Figure 4). They showed similar extent of CETP inhibitory activity.

However, in rHDL state, SCWA-rHDL and PCO-rHDL showed remarkably higher inhibitory activity against CETP than SCWA and PCO in ethanol (Figure 4). SCWA-rHDL (6 and 30 *μ*M SCWA) showed 26 and 45% inhibition, respectively, while PCO-rHDL (6 and 30 *μ*M PCO) showed 36% and 55% inhibition, respectively. Interestingly, PCO-rHDL showed 1.2-fold higher CETP inhibition activity than SCWA-rHDL.

### 3.5. PCO and SCWA Enhanced Growth of Brain Cell

**Figure 5:**
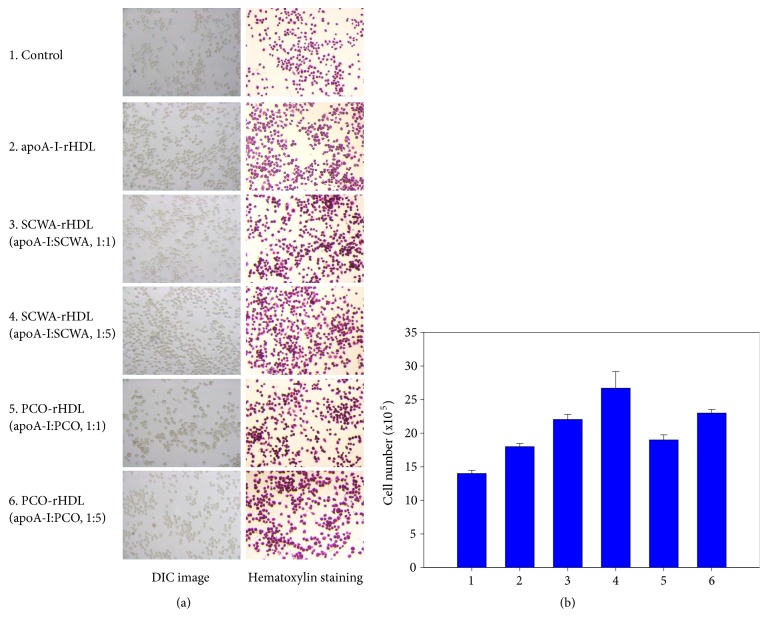
Enhancement of cell growth by rHDL containing SCWA and PCO in brain glial (BV-2) cells. Cell nucleus was visualized by hematoxylin staining in the presence of SCWA or PCO in rHDL. Photo 1, control; photo 2, apoA-I- rHDL; photo 3, SCWA-rHDL (1:1); photo 4, SCWA-rHDL (1:5); photo 5, PCO-rHDL (1:1); photo 6, PCO-rHDL (1:5).

SCWA-rHDL and PCO-rHDL treatment increased growth of brain glial cells in a dose-dependent manner as shown in Figure 5. Cells treated with rHDL showed 18.4 x 10^5^ cells from H&E staining, a 33% increase in cell numbers compared to PBS-treated cells (13.8 x 10^5^ cell), indicating that native-rHDL facilitated cell replication with cytoprotective properties. Interestingly, SCWA-rHDL and PCO-rHDL treatment increased more cell numbers. Treatment with SCWA-rHDL (1:1) and SCWA-rHDL (1:5) resulted in the highest cell number with 22.1 x 10^5^ cells and 26.7 x 10^5^ cells, respectively, corresponding to 9 and 45 *μ*M SCWA. Treatment with PCO-rHDL (1:1) and PCO-rHDL (1:5) resulted in 19.1 x 10^5^ cells and 23.1 x 10^5^ cells, respectively, corresponding to 9 and 45 *μ*M PCO.

### 3.6. Inhibition of oxLDL Uptake by PCO-rHDL and SCWA-rHDL

**Figure 6:**
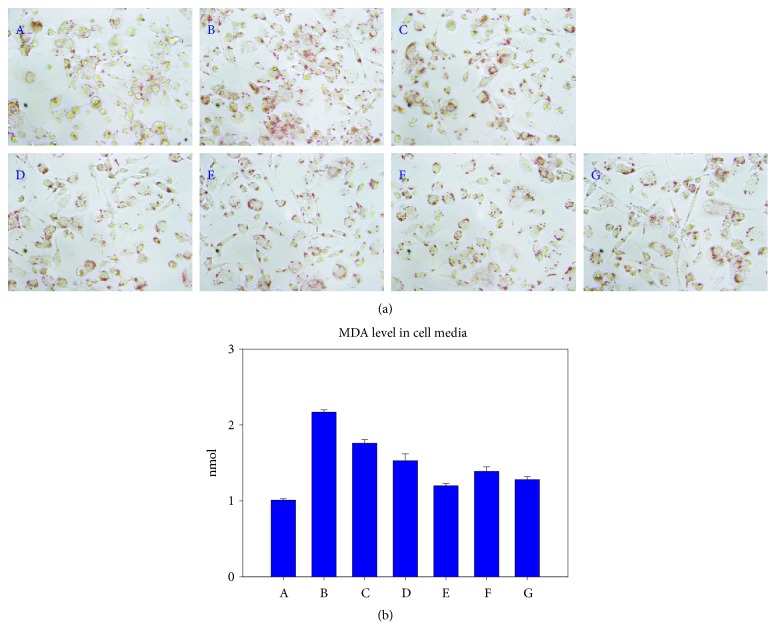
Antiatherogenic activity of PCO-rHDL or SCWA-rHDL under presence of oxLDL. Uptake of oxLDL into macrophages was visualized by oil red O staining. Photo A, blank control; photo B, oxLDL alone; photo C, oxLDL + rHDL; photo D, oxLDL + SCWA-rHDL (1:1); photo E, oxLDL + SCWA-rHDL (1:5); photo F, oxLDL + PCO-rHDL (1:1); photo G, oxLDL + PCO-rHDL (1:5).

As shown in Figure 6, compared to the PBS control (photo A), SCWA-rHDL and PCO-rHDL strongly facilitated uptake of oxLDL into macrophages, as visualized by oil red O staining (photo B). However, the phagocytosis of oxLDL was blocked by treatment with SCWA-rHDL or PCO-rHDL in a dose-dependent manner.

Quantification of oxidized species in cell media revealed that cells treated with oxLDL alone contained 2-fold more MDA than PBS-treated cells. As a control, rHDL-treated cells had 20% less MDA than those treated with oxLDL alone; however, SCWA-rHDL- or PCO-rHDL-treated cells contained 45% or 42% less MDA than cells treated with oxLDL alone.

### 3.7. Stimulation of Wound Healing

**Figure 7:**
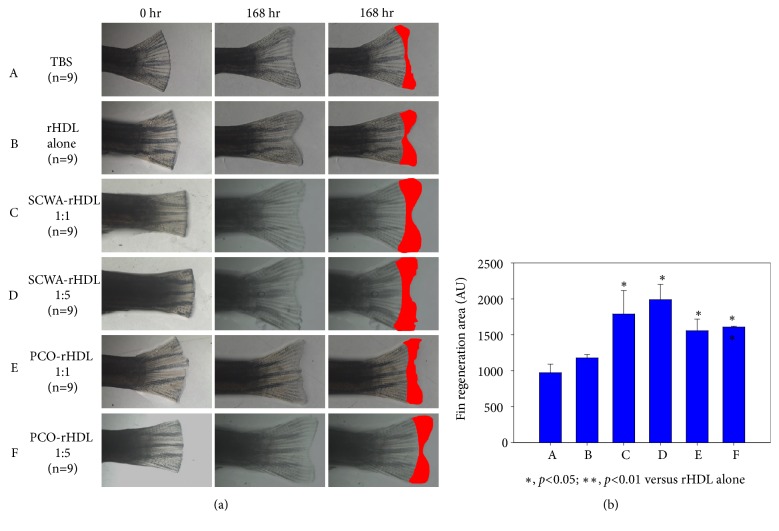
Wound healing activity of PCO-rHDL and SCWA-rHDL. Improvement of zebrafish fin regeneration by injection of reconstituted high-density lipoprotein (rHDL) containing either policosanol or SCWA. (a) Photo of tail fin regeneration upon injection of rHDL containing policosanol or SCWA with administration of normal diet. (b) Quantification of fin regeneration area at 168 h after the rHDL injection. Data shown are the mean±SD of three independent experiments (n=9). *∗*,* p*<0.05; *∗∗*, p<0.01.

With normal diet (ND) consumption, rHDL-injected group showed 1.2-fold higher tissue regeneration activity than the PBS-injected group over a period of 168 h (Figure 7(a)). The SCWA-rHDL- or PCO-rHDL-injected group showed 2.1-fold or 1.6-fold higher tissue regeneration activity, respectively, than the PBS control.

## 4. Discussion

Although PCO is known as a lipid-lowering agent that increases HDL-cholesterol levels [29] via CETP inhibition [13, 14], there have been many controversial reports about the cholesterol-lowering efficacy of policosanol [30, 31]. Berthold's group reported that policosanol (10, 20, 40, and 80 mg/day) was not effective in treating patients of hypercholesterolemia during 12-week consumption [32]. However, human studies with healthy Korean subjects suggested that 10-20 mg/day of policosanol could exert lipid-lowering and antihypertensive effects [12–14].

However, the physiological effect and molecular mechanism of SCWA are still not fully understood as it relates to lowering lipid levels. Both compounds have poor solubility in water and it has therefore been difficult to study their mechanism of action. To overcome this obstacle, we encapsulated PCO and SCWA into rHDL to solubilize them in aqueous buffer. Native HDL enhances endothelial function and the antioxidant capacity to inhibit LDL oxidation [33]. We presumed that enhancing HDL quality with these natural compounds could enhance its antioxidant (Figure 2), antiglycation (Figure 3), and antiatherogenic effects (Figure 6). The results of our* in vitro* study on suppression of LDL oxidation due to the antioxidant activity of PCO and SCWA (Figure 2) are in good agreement with previous clinical data [34].

Interestingly, as shown in Figure 4, Anacetrapib (MK-0859), a well-known CETP inhibitor, resulted in 41% and 62% inhibition* in vitro *at 6 and 30 *μ*M in EtOH, respectively. In ethanol, SCWA and PCO (final 6 and 30 *μ*M) also resulted in 15% and 26 % inhibition, respectively (Figure 4). They exhibited similar CETP inhibitory activity. The significant finding of this study was that both PCO and SCWA displayed potent CETP inhibitory activity in the context of CE transfer from HDL to LDL. CETP plays a critical role in lipid distribution among lipoproteins and is recognized as an atherogenic factor [35]. In the same context, the current results show that PCO-rHDL and SCWA-rHDL possess much higher CETP inhibitory activity than rHDL alone. This result suggests that PCO and SCWA might play a critical role in lipid homeostasis between HDL and LDL via exchange of CE and TG.

Regarding the detailed mechanism of CE transfer, a recent study reported that CETP binds to HDL via hydrophobic interactions because the HDL surface lipid curvature generates a hydrophobic environment [36]. Many reports on CETP inhibitors have concluded that the mechanism for CETP inhibition is based on competitive interactions with CETP and HDL [37]. It is plausible that long aliphatic chains in PCO and SCWA interfere with binding of CETP between LDL and HDL. The aliphatic chains of SCWA and PCO bind to a CE-binding site in the carboxyl terminus of CETP to form a ternary complex, as discussed in earlier studies [38]. The carboxyl terminus of CETP contains an active site and binding pocket for CE; a 12-amino-acid region (amino acids 453–476) forms an amphipathic *α*-helical region and helps in the transfer process [39]. A component of PCO and SCWA in HDL can bind to the amphipathic *α*-helix and block CE/TG transfer from HDL to LDL by interfering with CETP and LDL binding. In our current study, we showed that CETP inhibitory activity was enhanced by incorporation of PCO and SCWA into rHDL. Collectively, PCO and SCWA may interfere with HDL and LDL binding to CETP to form a hydrophobic channel.

Enhanced LDL catabolism and reduced TGs metabolism can be accelerated by inhibition of CETP. It has been asserted that CETP inhibition is a potent antiatherogenic strategy. Because of higher CETP activity, low HDL-C levels and prevalent dysfunctional HDL become risk factors for autoimmune disease with inflammation and other risk factors [40, 41]. The inhibition of CETP by PCO and SCWA reported in our study suggests that these natural compounds can lower inflammation and slow aging. Indeed, elevated cellular replication was observed (Figure 5) along with tissue regeneration (Figure 7).

Few studies on SCWA, which is a mixture of long chain aliphatic primary acids also known as D-003, have discussed its ability to lower lipid levels and its anti-inflammatory activities [18, 21, 42]. These natural compounds, PCO and D-003, inhibit cholesterol synthesis pathway by regulating HMG-CoA reductase, as reported by other groups [43, 44].

## 5. Conclusions

In conclusion, PCO and SCWA both enhance the beneficial functions of HDL to maximize its antioxidant, antiglycation, and antiatherosclerotic activities and the inhibition of CETP. These enhancements of HDL functionality by PCO and SCWA could exert antiaging and rejuvenation activity.

## Figures and Tables

**Table 1:** Characterization of rHDL containing PCO or SCWA by spectroscopic analysis.

		SCWA-rHDL			PCO-rHDL		
	Lipid free apoA-I	apoA-I:SCWA			apoA-I:PCO		
Molar ratio (apoA-I: SCWA or PCO)	-	1:0	1:1	1:5	1:0	1:1	1:5
Size (Å) ^a^	73	98	95	85	98	96	87
WMF (nm) ^b^	337	341	340	341	341	341	341

WMF, wavelengths of maximum fluorescence.

Molar ratio was 95:5:1:x for POPC:FC:ApoA-I:SCWA or PCO, where x is 0, 1, or 5.

^a^ Determined by 8% to 25% native-gradient gel electrophoresis with densitometric scanning analysis.

^b^ Determined by fluorospectroscopy to detect tryptophan (Trp) fluorescence.

## Data Availability

The table and figures data used to support the findings of this study are included within the article.
